# CRNDE: A valuable long noncoding RNA for diagnosis and therapy of solid and hematological malignancies

**DOI:** 10.1016/j.omtn.2022.03.006

**Published:** 2022-03-09

**Authors:** Xuefei Ma, Wen Jin, Chaoxian Zhao, Xuefeng Wang, Kankan Wang

**Affiliations:** 1 Department of Laboratory Medicine, Ruijin Hospital, Shanghai Jiao Tong University School of Medicine, Shanghai 200025, China; 2 Shanghai Institute of Hematology, State Key Laboratory of Medical Genomics, National Research Center for Translational Medicine, Ruijin Hospital, Shanghai Jiao Tong University School of Medicine, Shanghai 200025, China; 3 CNRS-LIA Hematology and Cancer, Sino-French Research Center for Life Sciences and Genomics, Ruijin Hospital, Shanghai Jiao Tong University School of Medicine, Shanghai 200025, China

**Keywords:** MT: Non-coding RNAs, long noncoding RNA, CRNDE, clinical value, molecular mechanism, solid tumor, hematological malignancy

## Abstract

Colorectal neoplasia differentially expressed (CRNDE) is an oncogenic long noncoding RNA (lncRNA). Increased CRNDE expression was initially discovered in colorectal cancer and then in a variety of solid tumors and hematological malignancies. CRNDE participates in multiple biological processes, such as cell proliferation, differentiation, migration, and apoptosis. CRNDE has been shown to modulate target gene expression through multiple mechanisms, including transcriptional regulation, post-transcriptional regulation, and competition for microRNA (miRNA) binding. In this review, we summarize the evidence that supports CRNDE in the diagnosis and prognosis predicting of cancers. The functional roles and molecular mechanisms of CRNDE are further described for major types of solid tumors and hematological malignancies. The therapeutic potential of CRNDE as a target for research and development is also discussed.

## Graphical abstract

**Figure:**
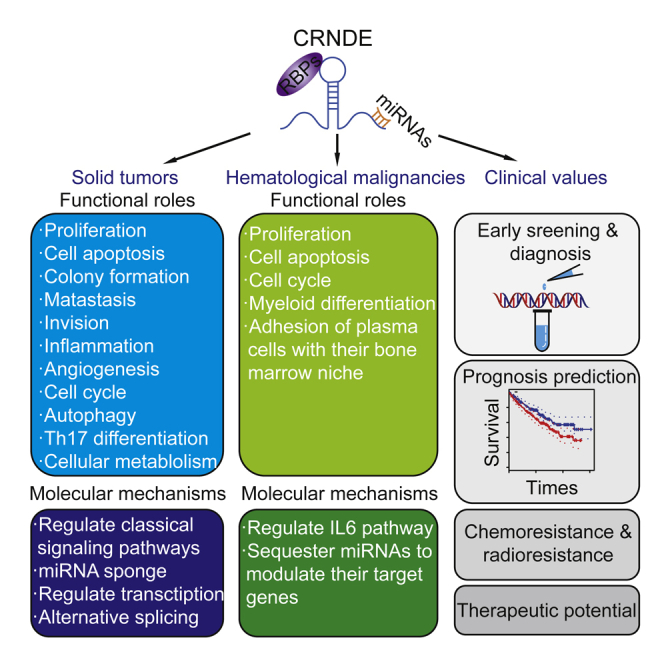

## Teaser

This review focuses on the clinical values of CRNDE in the diagnosis, prognosis predicting, and therapeutic potential as a target for research and development of cancers. The functional roles and molecular mechanisms of CRNDE are also summarized in solid tumors and hematological malignances.

Increasing numbers of long noncoding RNAs (lncRNAs; at least 200 nucleotides) have been shown to regulate gene expression and, by doing so, participate in a variety of physiological processes, including proliferation, differentiation, and apoptosis.^1^, ^2^, ^3^, ^4^ Aberrant expression of lncRNAs has been implicated in carcinogenesis, including both solid tumors and hematological malignancies,^2^, ^5^, ^6^, ^7^, ^8^, ^9^ at multiple mechanistic levels, including transcriptional and post-transcriptional regulation, chromatin conformation, pre-mRNA splicing, and competition for microRNA (miRNA) binding.^3^, ^4^, ^8^, ^9^, ^10^, ^11^

In this review, we focus on clinical and prognostic implications of lncRNA colorectal neoplasia differentially expressed (CRNDE) in both solid tumors and hematological malignancies. The functional roles and molecular mechanisms are also discussed.

## Overview of the genomic structure and functions of *CRNDE*

**Figure 1:**
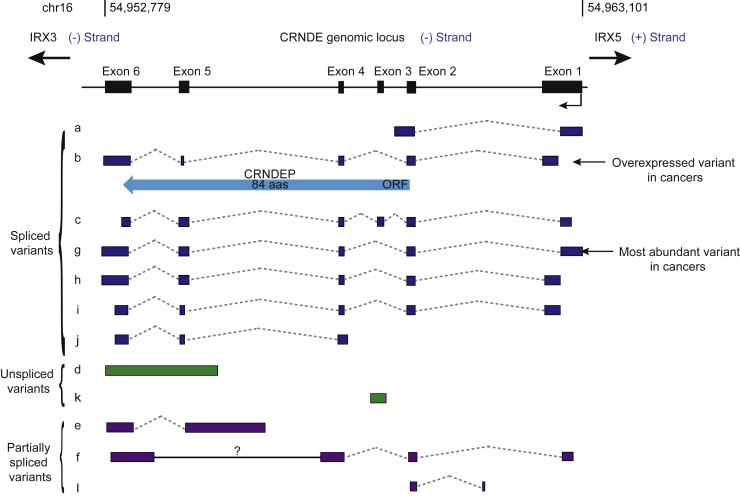
The *CRNDE* genomic locus The *CRNDE* genomic locus and the alternative splice variants are presented. Twelve CRNDE alternative splice variants are named from CRNDE-a to -l. The spliced, unspliced, and partially spliced variants are marked. The blue arrow represents the small peptide encoded by CRNDE. The cancer-related variants are also marked. The black, blue, green, and purple boxes represent exons. The solid and dashed lines represent introns.

The *CRNDE* gene spans from 54,952,779 to 54,963,101 on the reverse strand of chromosome 16.^12^ The upstream and downstream genes are the protein-coding genes iroquois homeobox 5 (*IRX5*) and iroquois homeobox (*IRX3*), respectively. The *CRNDE* gene encodes multiple splice variants.^13^ Based on the latest NCBI AceView database,^12^ there are at least 10 alternative transcript variants (Figure 1). Among them, CRNDE-a, -b, -c, -g, -h, -i, and -j are fully spliced variants, whereas CRNDE-e, -f, and -l are partially spliced variants.^14^ These alternative splice variants display distinct subcellular localization and tissue expression patterns.^13^, ^14^ For example, CRNDE-g and CRNDE-b are the most abundant variants in cancer cells.^14^ Subcellular fractionation analysis in CRNDE-overexpressing cell lines, including the HCT116 and HT29 colorectal cancer cell lines and NB4 acute promyelocytic leukemia (APL) cell line, has demonstrated that fully spliced variants are enriched in the cytoplasm, whereas retained intron transcripts are enriched in the nucleus.^15^, ^16^, ^17^ However, despite the predominant location of fully spliced CRNDE variants in the cytoplasm, one of the fully spliced variants, CRNDE-b (GenBank: FJ466686), can encode an 84-amino-acid peptide, named CRNDEP, which in turn is mainly located in the nucleus and upregulated in highly proliferating tissues, such as the parabasal layer of spermatocytes or intestinal crypts.^18^

**Figure 2:**
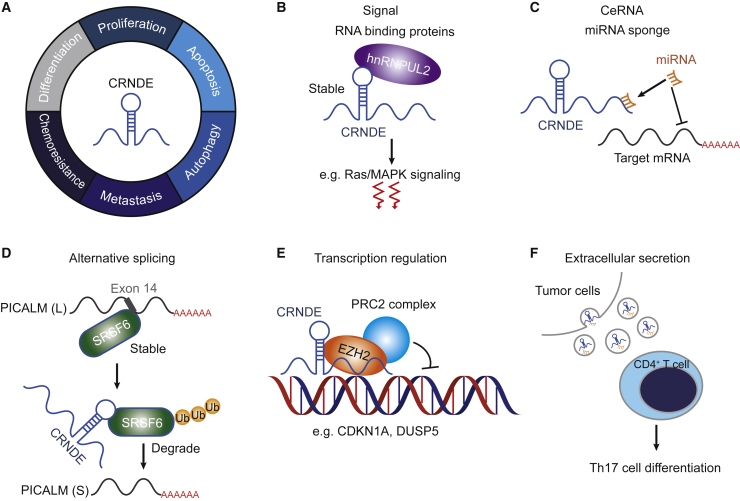
Functional mechanisms of CRNDE in carcinogenesis (A) The functions of CRNDE in cancer development.^3^, ^16^, ^19^, ^20^, ^21^. (B–F) CRNDE regulatory mechanisms. (B) CRNDE can form a complex with hnRNPUL2 in the cytoplasm and activate Ras/MAPK signaling pathways.^19^ (C) CRNDE has several miRNA binding sites and serves as a competing endogenous RNA (ceRNA) to sponge miRNAs, thereby impeding their inhibitory effect on the expression of target mRNAs.^22^, ^23^, ^24^ (D) CRNDE modulates alternative splicing events by interacting with the splicing factor SRSF6 to induce proteasome ubiquitination (Ub)-dependent SRSF6 degradation.^3^ The low expression of SRSF6 suppresses a short (S) to long (L) isoform switch of PICALM, thereby increasing the production of the exon 14 skip variant of PICALM.^3^ (E) CRNDE recruits epigenetic regulator EZH2 to inhibit the transcription of *CDKN1A* and *DUSP5*.^25^ (F) CRNDE can be transferred to CD4^+^ T cells by tumor exosomes to induce the Th17 cell differentiation.^26^

CRNDE, as a multifunctional lncRNA, plays prominent roles in cancers, including both solid tumors and hematological malignancies. Functional investigations reveal that CRNDE can participate in cell cycle, differentiation, proliferation, metastasis, autophagy, adhesion, and apoptosis. The mechanisms of CRNDE in gene regulating include sponging miRNAs and proteins to modulate downstream target genes or pathways, participating alternative splicing, and gene transcriptional regulation (Figure 2). The details of the functional roles of CRNDE in cancers are summarized in this article.

## Expression patterns and prognostic values of CRNDE in solid and hematological malignancies

**Figure 3:**
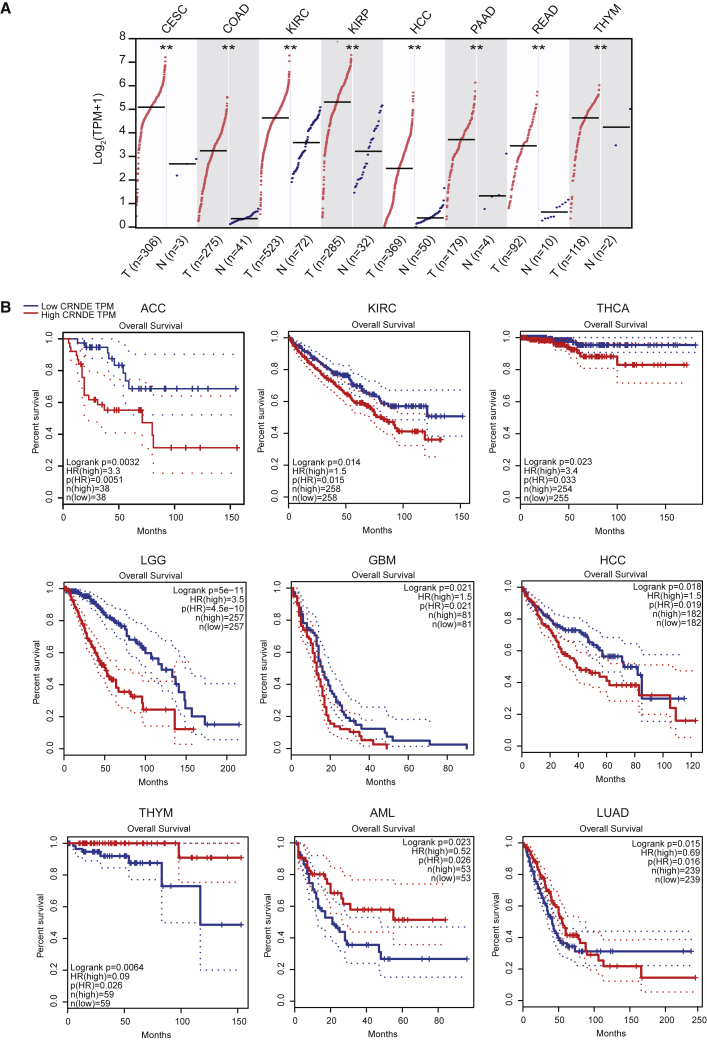
Expression and prognostic analysis of CRNDE in different types of cancers (A) CRNDE expression is significantly upregulated in eight types of cancers compared with corresponding normal tissues. ∗∗p < 0.01. The full cancer names are listed in Table 1. (B) The overall survival curves of CRNDE depict the prognostic significance (Log-rank test, p < 0.05) in nine cancers using GEPIA.^29^ The Cox proportional hazard ratio (HR) is presented in the survival plot. The dotted lines show the 95% confidence interval (CI) information.

CRNDE was originally identified to be overexpressed in colorectal cancer (CRC) and subsequently found to be upregulated in many solid tumors as well as hematological malignancies.^13^, ^14^, ^27^, ^28^ Analysis of CRNDE expression in 23 types of cancer using the GEPIA web server (http://gepia.cancer-pku.cn),^29^ based on The Cancer Genome Atlas (TCGA) database, shows that CRNDE is overexpressed in eight types of cancer (cervical squamous cell carcinoma and endocervical adenocarcinoma, colon adenocarcinoma, kidney renal clear cell carcinoma, kidney renal papillary cell carcinoma, hepatocellular carcinoma, pancreatic adenocarcinoma, rectum adenocarcinoma, and thymoma [THYM]), compared with corresponding normal controls (Figure 3A). These indicate that CRNDE is a potential biomarker for cancer diagnosis.

CRNDE expression could be used to predict prognosis in cancer patients.^3^, ^28^, ^30^ Analysis of the TCGA database has shown an association between high CRNDE expression with unfavorable prognosis in six types of cancer: adrenocortical carcinoma, kidney renal clear cell carcinoma, thyroid carcinoma, low-grade glioma, glioblastoma multiforme, and hepatocellular carcinoma (Table 1; Figure 3B). A meta-analysis indicated that CRNDE overexpression is associated with lymph node metastasis and advanced tumor-node-metastasis (TNM) stage.^31^, ^32^, ^33^ These suggest that high CRNDE expression can be a reliable indicator for poor survival in these types of cancer. Intriguingly, high CRNDE expression has also been associated with good survival in THYM and acute myeloid leukemia (AML) (Table 1 and Figure 3B). Although the reason is not very clear, we hypothesize that the therapeutic efficiency and sensitivity and the immune infiltration level may be higher in THYM and AML than in other cancers. The survival prediction power of CRNDE expression in patients with lung adenocarcinoma (LUAD) seems complicated, as shown by a relatively favorable outcome within 90 months and an unfavorable outcome beyond 90 months (Table 1 and Figure 3B). In a previous study, high CRNDE expression is associated with adverse prognosis in LUAD.^34^ This may be due to the use of clinical data from different sources.

**Table 1:** The overall survival analysis of patients with different types of cancer according to CRNDE expression using TCGA RNA-Seq data

Cancer Types	Full Names of Cancers	N (High)	N (Low)	Log-Rank p Value	HR (High)	p (HR)
ACC	adrenocortical carcinoma	38	38	0.0032	3.3	0.0051
BLCA	bladder urothelial carcinoma	201	201	0.21	1.2	0.21
BRCA	breast invasive carcinoma	535	535	0.26	1.2	0.26
CESC	cervical squamous cell carcinoma and endocervical adenocarcinoma	146	146	0.74	0.92	0.74
CHOL	cholangiocarcinoma	18	18	0.11	2.2	0.11
COAD	colon adenocarcinoma	135	135	0.52	0.86	0.52
DLBCL	diffuse large B cell lymphoma	23	23	0.34	2	0.35
ESCA	esophageal carcinoma	91	91	0.95	0.98	0.93
GBM	glioblastoma multiforme	81	81	0.021	1.5	0.021
HNSC	head and neck squamous cell carcinoma	259	259	0.59	1.1	0.59
KICH	kidney chromophobe	32	32	0.25	2.2	0.27
KIRC	kidney renal clear cell carcinoma	258	258	0.014	1.5	0.015
KIRP	kidney renal papillary cell carcinoma	141	141	0.33	0.74	0.34
AML	acute myeloid leukemia	53	53	0.023	0.52	0.026
LGG	low-grade glioma	257	257	5.00 × 10^−11^	3.5	4.50 × 10^−10^
HCC	hepatocellular carcinoma	182	182	0.018	1.5	0.019
LUAD	lung adenocarcinoma	239	239	0.015	0.69	0.016
LUSC	lung squamous cell carcinoma	241	241	0.99	1	0.99
MESO	mesothelioma	41	41	0.91	1	0.9
OV	ovarian serous cystadenocarcinoma	212	212	0.74	0.96	0.74
PAAD	pancreatic adenocarcinoma	89	89	0.15	1.3	0.16
PCPG	pheochromocytoma and paraganglioma	91	91	0.58	1.6	0.58
PRAD	prostate adenocarcinoma	246	246	0.42	1.7	0.43
READ	rectum adenocarcinoma	46	46	0.98	0.99	0.98
SARC	sarcoma	131	131	0.59	0.9	0.59
SKCM	skin cutaneous melanoma	228	229	0.32	1.1	0.32
STAD	stomach adenocarcinoma	192	192	0.6	0.92	0.6
TGCT	testicular germ cell tumors	67	68	0.47	0.44	0.48
THCA	thyroid carcinoma	254	255	0.023	3.4	0.033
THYM	thymoma	59	59	0.0064	0.09	0.026
UCEC	uterine corpus endometrial carcinoma	86	86	0.18	0.62	0.18

HR, hazard ratio.

## CRNDE acts as an oncogenic lncRNA in solid tumors

CRNDE is implicated in diverse cellular functions, including serving as a sponge to sequester miRNAs and proteins to regulate signaling pathways in cancer development.^19^, ^20^, ^28^, ^35^, ^36^, ^37^ In this part, we discuss the clinical values and functional roles of CRNDE in different solid tumors (Table 2).

**Table 2:** The clinical values and functions of CRNDE in solid and hematological malignancies

Cancer types		Clinical values	Functions	Molecular mechanisms	References
Solid tumors	CRC	upregulated expression in CRC and plasma exosomes, unfavorable outcomes, drug resistance of oxaliplatin	proliferation, metastasis, invasion, chemoresistance, cellular metabolism, Th17 cell differentiation	miR-181a-5p/Wnt/β-catenin pathway, miR-136/E2F1, insulin/IGF signaling pathway, miR-217/TCF7L2/Wnt/β-catenin pathway, EZH2/DUSP5/CDKN1A, hnRNPUL2/Ras/MAPK pathway, RORγt	19, 20, 22, 25, 26, 38, 39
	GM	upregulated expression in glioma tissues, higher risk of WHO grade, higher recurrence rate, and poor overall survival	cell growth, invasion, inflammation, proliferation, migration, apoptosis	miR-186/XIAP/PAK5, toll-like receptor pathway, mTOR and EGFR signaling pathways, miR-384/PIWIL4/STAT3, miR-136-5p/Bcl-2/Wnt	40, 41, 42, 43, 44, 45
	HCC	upregulated expression in HCC tissues, an effective diagnostic biomarker with high sensitivity and specificity, chemoresistance of adriamycin and cisplatin, poor prognosis	proliferation, migration, invasion, epithelial-mesenchymal transition, chemoresistance, angiogenesis	miR-384/NF-κB and p-AKT, miR-217/MAPK1, miR-203/BCAT1, miR-136-5p/IRX5, miR-337-3p/SIX1, directly bound EZH2/SUZ12/SUV39H1 to regulate CELF2 and LATS2, miR-33a-5p/CDK6, miR-539-5p/POU2F1, miR-203/VEGFA, PI3K/AKT/β-catenin pathway, Wnt/β-catenin signaling pathway	23, 28, 46, 47, 48, 49, 50, 51, 52, 53, 54
	LC	upregulated expression in lung cancer tissues, poor differentiation, classification of TNM stages, lymph node metastasis, radiotherapy resistance, and a shorter overall survival	proliferation, apoptosis, colony formation, migration, invasion	miR-641/CDK6, miR-338-3p, PRC2/EZH2/P21, PI3K/AKT signaling pathway	34, 37, 55, 56
	CC	upregulated expression in cervical cancer tissues, negative correlated with overall survival	proliferation, apoptosis, cell growth, migration and invasion	PUMA, miR-183/CCNB1, miR-4262/ZEB1, PI3K/AKT signaling pathway	21, 57, 58, 59
	BC	upregulated expression in BC tissues, larger tumor size, advanced TNM stage, and unfavorable prognosis	proliferation, migration, invasion	miR-136/Wnt/β-catenin signaling pathway	^60^
	TSCC	upregulated expression in TSCC tissues	proliferation, cell cycle, invasion	miR-384	^61^
	PC	upregulated expression in pancreatic cancer tissues, poor clinicopathological characteristics, and shorter overall survival	proliferation, metastasis,	miR-384/IRS1	^24^
	GC	low CRNDE suppresses the response to 5-FU/oxaliplatin-based chemotherapy	autophagy	SRSF6-mediated alternative splicing of PICALM	^3^
	PTC	upregulated expression in PTC tissues	proliferation, migration, invasion	miR-384/PTN	^62^
	MB	upregulated expression in medulloblastoma tissues, resistance to chemotherapeutics	cell viability, colony formation, apoptosis, migration and invasion, repression of CRNDE increases chemosensitivity	miR-29c-3p	^63^
	RCC	upregulated expression in RCC tissues	viability, migration, invasion of RCC	miR-136-5p	^64^
	PCA	upregulated expression in PCA tissues, poor outcomes	proliferation, migration, invasion	miR-101/Rap1A	^65^
	OS	upregulated expression in OS tissues	proliferation, migration, invasion, cell cycle, epithelial-mesenchymal transition, differentiation	Notch1 signaling pathway, GSK-3β/Wnt/β-catenin signaling pathway	36, 66, 67
	MEL	upregulated expression in melanoma tissues	proliferation, metastasis	miR-205/CCL18	^68^
Hematological malignancies	MM	upregulated expression in MM, poor prognosis	proliferation, cell cycle, apoptosis, affects the adhesion of tumor cells with their bone marrow niche	miR-451, IL6R and CDH2/IL6 signaling	69, 70
	AML	upregulated expression in the APL and *NPM1*-mutant AML BMs	proliferation, differentiation	miR-181/NOTCH2	14, 16
	ALL	upregulated expression in the BM of BCP-ALL	proliferation, apoptosis	miR-345-5p/CREB	14, 71
	CLL	downregulated expression in the BM of CLL	proliferation, apoptosis	miR-28/NDRG2	14, 72

CRC, colorectal cancer; GM, gliomas; HCC, hepatocellular carcinoma; LC, lung cancer; CC, cervical cancer; BC, breast cancer; TSCC, oral/tongue squamous cell carcinoma; PC, pancreatic cancer; GC, gastric cancer; PTC, papillary thyroid carcinoma; MB, medulloblastoma; RCC, renal cell carcinoma; PCA, prostate cancer; OS, osteosarcoma; MEL, melanoma; MM, multiple myeloma; AML, acute myeloid leukemia; ALL, acute lymphocytic leukemia; CLL, chronic lymphocytic leukemia, BM, bone marrow. TCF7L2, transcription factor 7-like 2; DUSP5, dual specificity phosphatase 5; CDKN1A, cyclin dependent kinase inhibitor 1A; hnRNPUL2, heterogeneous nuclear ribonucleoprotein U like 2; RORγt, RAR-related orphan receptor γt; XIAP, X-linked inhibitor of apoptosis; PAK5, p21 (RAC1) activated kinase 5; PIWIL4, piwi like RNA-mediated gene silencing 4; MAPK1, mitogen-activated protein kinase 1; BCAT1, branched chain amino acid transaminase 1; SIX1, SIX homeobox 1; CDK6, cyclin dependent kinase 6; IRX5, iroquois homeobox 5; SUZ12, suppressor of zeste 12; CELF2, CUGBP elav-like family member 2; POU2F1, POU class 2 homeobox 1; VEGFA, vascular endothelial growth factor a; PUMA, p53 upregulated modulator of apoptosis; CCNB1, cyclin b1; IRS1, insulin receptor substrate 1; SRSF6, serine and arginine rich splicing factor 6; PICALM, phosphatidylinositol binding clathrin assembly protein; PTN, pleiotrophin; Rap1A, ras-related protein 1A; GSK-3β, glycogen synthase kinase-3β; CCL18, C-C motif chemokine ligand 18; CREB, cyclic AMP response element-binding protein; NDRG2, NDRG family member 2.

### Colorectal cancer

The incidence of CRC has been increasing dramatically year by year. Currently, CRC is the fourth leading cause of cancer-related mortality globally.^73^ Early diagnosis, prognostic assessment, targeted therapy, and mechanical research of tumorigenesis are of particular importance to CRC.^74^ Overexpressed CRNDE in blood and CRC tissues has been shown to be a predictor for the early screening and diagnosis of CRC.^30^ For example, one of these transcripts, CRNDE-h, is highly expressed in plasma exosomes of CRC patients and significantly associated with unfavorable outcomes.^30^ In a microarray analysis of 522 colorectal tissue specimens, CRNDE is overexpressed in more than 90% of the CRC samples compared with non-cancerous tissues.^13^ CRNDE is also involved in resistance to chemotherapy. Several studies indicate that CRNDE knockdown can reduce chemoresistance of CRC cells to oxaliplatin, while CRNDE overexpression increases the anti-apoptosis capability of CRC cells during oxaliplatin treatment.^20^, ^38^ These results suggest that CRNDE is a promising biomarker of CRC for diagnosis, progression, and therapy.

CRNDE serves as an oncogenic lncRNA promoting CRC progression by modulating critical signaling pathways. For example, upregulated CRNDE accelerates proliferation and metastasis of CRC cells by regulating Wnt/β-catenin signaling pathways.^20^, ^22^ CRNDE also forms a complex with heterogeneous nuclear ribonucleoprotein U like 2 (hnRNPUL2) in the cytoplasm and activates Ras/MAPK signaling pathways, thereby accelerating CRC cell proliferation and migration.^19^ Nuclear transcripts of CRNDE could promote the metabolic changes of glucose and lipid by affecting insulin/IGF signaling pathways, in a pattern of the Warburg effect.^39^ At an epigenetic level, CRNDE modulates transcription of the proliferation-associated genes by binding to histone modification enzymes. CRNDE binding to EZH2, a core component of the polycomb repressive complex 2 (PRC2), epigenetically inhibits the expression of dual-specificity phosphatase 5 (DUSP5) and cyclin-dependent kinase inhibitor 1A (CDKN1A), thereby promoting CRC development.^25^ CRNDE could also serve as a competing endogenous RNA (ceRNA) to regulate mRNA expression. For example, CRNDE increases the expression of transcription factor 7-like 2 (TCF7L2) and activates Wnt/β-catenin signaling by competitively binding with miR-217.^22^ Recent study shows that CRNDE also influences the tumor immune microenvironment. CRNDE-h in exosomes promotes Th17 cell differentiation by repressing the E3 ubiquitin ligase Itch-mediated ubiquitination and degradation of RAR-related orphan receptor γt (RORγt).^26^ Together, these findings indicate that CRNDE promotes CRC progression via multiple mechanisms.

### Gliomas

Gliomas (World Health Organization [WHO] classification: I, II, III, and IV grades) are the most malignant and aggressive tumors of primary intracranial carcinoma, among which glioblastoma multiforme (grade IV) accounts for more than 50% of malignant gliomas with a dreadful overall survival.^75^ CRNDE expression has been shown to be closely associated with the development of gliomas and is useful for prognosis monitoring for gliomas in many studies.^14^, ^76^, ^77^ First, CRNDE is upregulated in glioma tissues compared with non-tumor samples.^14^ More importantly, higher CRNDE expression is observed more frequently in advanced gliomas and associated with larger tumor size and higher WHO grade.^76^ Second, CRNDE upregulation is also detected in recurrent glioma patients.^76^, ^77^ The survival analysis based on a cohort of 164 glioma patients shows that CRNDE is an independent risk of poor prognosis in patients with gliomas.^76^

Several mechanisms have been proposed for the oncogenic action of CRNDE in gliomas. First, CRNDE regulates pivotal signaling pathways of tumorigenesis in gliomas. For example, CRNDE triggers inflammation to modulate carcinogenesis by activating the toll-like receptor signaling pathway.^40^ CRNDE also promotes cell proliferation and growth of glioma cells by regulating the epidermal growth factor receptor (EGFR) and mammalian target of rapamycin (mTOR) signaling pathways.^41^, ^42^ Second, CRNDE acts as an miR-186 sponge to upregulate the expression of X-linked inhibitor of apoptosis (XIAP) and p21 (RAC1) activated kinase 5 (PAK5), thereby modulating the downstream apoptosis pathway.^43^ In another study, CRNDE facilitates glioma cell proliferation and migration and represses apoptosis by regulating the expression of miR-384 and its target piwi-like RNA-mediated gene silencing 4 (PIWIL4).^44^ CRNDE also promotes glioma pathogenesis by preventing miR-136-5p-mediated downregulation of Bcl-2 and Wnt2.^45^ Together, CRNDE can regulate the development of gliomas.

### Hepatocellular carcinoma

Hepatocellular carcinoma (HCC) accounts for more than 80% of primary liver cancers.^78^ A large number of HCC patients are diagnosed in advanced stages, thereby losing the chance of suitable radical treatment.^79^ Therefore, monitoring of HCC high-risk patients and screening reliable biomarkers of HCC in the early stage are essential to improve the outcome of HCC patients.^80^, ^81^ In a high-throughput analysis of RNA sequencing (RNA-seq) data from 23 liver tissues encompassing controls, cirrhotic tissues, and HCCs, and in a comprehensive analysis of six datasets encompassing HCC and matched paracancerous tissues from the Gene Expression Omnibus (GEO) database, CRNDE overexpression possesses significant diagnostic and prognostic values.^82^, ^83^ Integrative analysis of TCGA and GEO databases has shown that CRNDE is an effective diagnostic biomarker with high sensitivity and specificity in HCC.^33^ Moreover, CRNDE participates in chemoresistance of HCC. Knockdown of CRNDE enhances adriamycin and cisplatin sensitivity by regulating epigenetic suppression of CUGBP elav-like family member 2 (CELF2) and large tumor suppressor kinase 2 (LATS2) in HCC cell lines.^28^ These findings suggest that CRNDE overexpression could be used in the diagnosis and prognosis prediction and treatment response monitoring.

CRNDE promotes HCC cell proliferation via the PI3K/AKT pathway and regulates the epithelial-mesenchymal transition of HCC cells by activating Wnt/β-catenin pathways.^46^, ^47^ CRNDE also modulates miRNAs and its downstream targets in HCC, such as the miR-384/nuclear factor (NF)-κB and p-AKT axis, the miR-217/mitogen-activated protein kinase 1 (MAPK1) axis, the miR-203/branched chain amino acid transaminase 1 (BCAT1) axis, the miR-203/vascular endothelial growth factor A (VEGFA) axis, the miR-136-5p/IRX5 axis, miR-337-3p/SIX homeobox 1 (SIX1) axis, the miR-33a-5p/cyclin-dependent kinase 6 (CDK6) axis, and the miR-539-5p/POU class 2 homeobox 1 (POU2F1) axis.^23^, ^48^, ^49^, ^50^, ^51^, ^52^, ^53^, ^54^

### Other solid tumors

In addition to the solid tumors discussed above, CRNDE is also upregulated and serves as an oncogenic lncRNA in other types of solid tumors, including lung cancer, cervical cancer, breast cancer, oral/tongue squamous cell carcinoma, pancreatic cancer, gastric cancer, papillary thyroid carcinoma, osteosarcoma, and melanoma. In lung cancer, high expression of CRNDE is associated with poor differentiation, advanced TNM stage, lymph node metastasis, radiotherapy resistance, and shorter overall survival.^34^ Mechanistically, CRNDE binds to PRC2 and recruits its core component EZH2 to repress p21 transcription, finally increasing the radioresistance of LUAD cells.^34^ In non-small cell lung cancer, CRNDE knockdown inhibits proliferation and promotes apoptosis by regulating the miR-641/CDK6 axis; represses colony formation, migration, and invasion via sponging miR-338-3p *in vitro*; and decreases the xenograft tumor volume and weight *in vivo*.^55^, ^56^ In cervical cancer, CRNDE binds to p53 upregulated modulator of apoptosis (PUMA) to enhance cervical cancer cell growth.^21^ CRNDE also modulates the expression of cyclin b1 (CCNB1) through sponging miR-183 to induce cell migration and invasion.^57^ Besides, CRNDE activates the PI3K/AKT pathway to promote proliferation and inhibit apoptosis in cervical cancer, and the high expression level of CRNDE is negatively correlated with overall survival.^58^ In breast cancer, CRNDE regulates Wnt/β-catenin pathways by serving as an miRNA sponge of miR-136.^60^ In gastric cancer, CRNDE participates in autophagy regulation and decreases chemoresistance through serine- and arginine-rich splicing factor 6 (SRSF6)-mediated alternative splicing of phosphatidylinositol binding clathrin assembly protein (PICALM).^3^ In osteosarcoma, CRNDE can promote cell proliferation, invasion, and migration by enhancing the activity of Notch1 signaling pathways and Wnt/β-catenin pathways.^36^, ^66^ CRNDE knockdown suppresses the tumor growth of osteosarcoma in the nude mice by inhibiting the mRNA expression of Notch1, Jag1, N-cadherin, and vimentin, and increased the mRNA expression of E-cadherin.^66^ In melanoma, CRNDE promotes proliferation and metastasis by competitively binding to miR-205 and targeting C-C motif chemokine ligand 18 (CCL18).^68^ In addition to HCC and gliomas, CRNDE also serves as an miRNA sponge of miR-384 to regulate disease progression in tongue squamous cell carcinoma, pancreatic cancer, and papillary thyroid cancer.^24^, ^61^, ^62^ Recently, the oncogenic roles of CRNDE via regulating miRNAs were also demonstrated in medulloblastoma, renal cell carcinoma, and prostate cancer.^63^, ^64^, ^65^ The detailed mechanisms still need to be further explored. Taken together, these studies demonstrate that CRNDE promotes the initiation, progression, and chemoresistance of many solid tumors.

## The emerging roles of CRNDE in hematological malignancies

The emerging significance of CRNDE for hematological malignancies, both as a biomarker for diagnosis and as a target for developing therapies, has generated broad interest. Aberrant CRNDE expression has been found in a variety of hematological malignancies (Table 2). Analysis of published microarray data in the GEO database showed upregulation of CRNDE in many types of hematological malignancies, including AML, acute lymphoblastic leukemia (ALL), chronic myeloid leukemia (CML), and myelodysplastic syndromes (MDSs).^14^ Meng et al. have demonstrated higher CRNDE expression in multiple myeloma (MM) than in the healthy control samples.^69^ DNA methylation and gene expression analysis provides evidence that, compared with CD19^+^-sorted B cells, low CRNDE expression in chronic lymphocytic leukemia (CLL) is correlated with the hypermethylation on its promoter region.^84^ These findings suggest that abnormally expressed CRNDE could be used as a broad biomarker in multiple hematological malignancies.

Abnormally high expression of CRNDE in leukemia has been found to be reduced upon the treatment of differentiation-inducing agents.^14^ For example, in AML cell line THP1, the expression of CRNDE is decreased by phorbol 12-myristate 13-acetate (PMA), a common drug to induce terminal monocyte-macrophage differentiation.^14^ Besides, after CML treatment with imatinib, downregulation of CRNDE expression is detected in bone marrow samples of CML patients.^14^ These findings imply that CRNDE downregulation may participate in differentiation therapy, although there is no experimental evidence that CRNDE is a direct target of these differentiation-inducing anti-cancer drugs. Additional experiments that manipulate CRNDE expression more specifically are needed to examine the therapeutic role of CRNDE.

### AML

AML is driven by initiating genetic events and requires secondary events. These events include gene mutations, aberrant expression of oncogenes and tumor suppressor genes, and epigenetic alterations.^85^, ^86^, ^87^ Similar to the observations in solid tumors, previous studies have reported that overexpressed CRNDE promotes the malignant progression in AML cell line U937.^88^ A recent study from our laboratory has indicated that CRNDE might serve as a cooperative event with PML/RARα or *NPM1* mutations to promote the progression of APL or *NPM1*-mutant AML.^16^ We have found that the expression of CRNDE is elevated in patients with APL and *NPM1*-mutant AML by analyzing transcriptome data from a large cohort of AML samples and normal controls.^16^ The oncogenic role of CRNDE in APL and *NPM1*-mutant AML is associated with differentiation block and/or cell proliferation.^16^ CRNDE knockdown can reduce the leukemogenic potential of PML/RARα-positive cells and prolongs the survival of APL mice.^16^ Further mechanistic investigations have demonstrated that CRNDE directly binds to the miR-181 family and thereby regulates NOTCH2 to exert its oncogenic role.^16^

### Lymphocytic leukemia

Lymphocytic leukemia is classified into ALL and CLL, according to the severity of the disease and the degree of leukemia cell differentiation. CRNDE expression is elevated in ALL but decreased in CLL.^14^ Ni et al. demonstrated that the DNA hypermethylation status is associated with CRNDE downregulation in CLL.^72^ However, the epigenetic state of the *CRNDE* gene in ALL remains unknown. We speculate that the differential expression of CRNDE between ALL and CLL may be associated with the different degrees of the lymphocyte differentiation.

Interestingly, CRNDE produces the opposite impact on cell proliferation in ALL versus CLL. CRNDE promotes the progression of leukemic cells in B cell precursor ALL (BCP-ALL), but inhibits the disease progression in CLL.^71^, ^72^ Mechanistically, in BCP-ALL, CRNDE targets the miR-345-5p/cyclic AMP response element-binding protein (CREB) axis to promote cell growth.^71^ In CLL, CRNDE regulates the expression of NDRG family member 2 (NDRG2) via sponging miR-28 to suppress proliferation and boost apoptosis of MEG1 and HG3 cells.^72^ The distinct effect of CRNDE on proliferation in ALL and CLL implies that expression level of CRNDE needs to be maintained at an appropriate level during lymphocytic differentiation.

### MM

MM is one of the most common hematopoietic diseases, characterized by abnormal proliferation of immunoglobulin-secreting plasma B cells in the bone marrow, accounting for about 10% of hematological malignancies.^89^ Despite advances in treatment and improvement in patient survival, the relapse rate remains high, and MM is thus still regarded as an incurable disease.^90^ Dysregulation of the interleukin 6 (IL6) signaling pathway plays a critical role in MM progression, relapse, and dexamethasone resistance, and is the most common therapeutic target in developing efforts.^91^ A recent study suggested that CRNDE promotes MM cell proliferation likely by regulating IL6 receptor (IL6R) expression.^70^ Also, CRNDE knockdown increases sensitivity to dexamethasone through impairing IL6 signaling.^70^ These results encourage development efforts in targeting CRNDE expression in MM.

The bone marrow microenvironment critically affects the invasion and progression of MM. CRNDE could affect the bone marrow niche and alter the adhesion of MM tumor cells.^70^ High CRNDE expression induces the cell adhesion molecule N-cadherin CDH2 expression in plasma cells of MM patients, thereby increasing the MM plasma cell adhesion to bone marrow stromal cells.^70^ The adhesive properties of MM plasma cells influenced by CRNDE can produce positive feedback in enhancing IL6 signaling.^70^ An additional function of CRNDE is inducing anti-apoptosis capability and cell-cycle arrest in the G0/G1 phase of MM cells.^69^ CRNDE mediates tumorigenesis in MM partially through targeting miR-451.^69^

## Conclusions and perspectives

In this article, we review the clinical implications of CRNDE in solid tumors and hematological malignancies. CRNDE overexpression is correlated with disease progression and could be used as a biomarker for most caner types. CRNDE acts as a sponge for miRNAs or proteins to regulate multiple cancer-associated genes and pathways, including cell cycle, differentiation, proliferation, migration, autophagy, adhesion, and apoptosis. CRNDE could potentially be used in diagnosis, prognosis prediction, and treatment response monitoring, but requires much more evidence for eventual implementation. Limited studies also suggest CRNDE could be explored as a target for developing novel treatments for certain types of malignancy.

The precise mechanisms by which CRNDE contributes to cancer development remain largely unclear. First, available evidence indicates that CRNDE produces many biological actions by serving as an miRNA sponge. Binding to proteins is an alternative route of action. The mechanism by which CRNDE works in this way also needs extensive investigation. Second, epigenetic events, most notably DNA methylation, have also been implicated, but again with only limited evidence. Third, the functional role of tissue-specific expression of multiple CRNDE splice variants requires further study. It seems that CRNDE transcripts may produce biological action by serving as both lncRNA and CRNDE-encoded small peptides. The function of CRNDE-encoded small peptides is a brand-new research field for the future.

## References

[1] Lu X., Qiao L., Liu Y. (2020). Long noncoding RNA LEF1-AS1 binds with HNRNPL to boost the proliferation, migration, and invasion in osteosarcoma by enhancing the mRNA stability of LEF1. J. Cell Biochem..

[2] Gandhi M., Gross M., Holler J.M., Coggins S.A., Patil N., Leupold J.H., Munschauer M., Schenone M., Hartigan C.R., Allgayer H. (2020). The lncRNA lincNMR regulates nucleotide metabolism via a YBX1 - RRM2 axis in cancer. Nat. Commun..

[3] Zhang F., Wang H., Yu J., Yao X., Yang S., Li W., Xu L., Zhao L. (2021). LncRNA CRNDE attenuates chemoresistance in gastric cancer via SRSF6-regulated alternative splicing of PICALM. Mol. Cancer.

[4] Singh N., Ramnarine V.R., Song J.H., Pandey R., Padi S.K.R., Nouri M., Olive V., Kobelev M., Okumura K., McCarthy D. (2021). The long noncoding RNA H19 regulates tumor plasticity in neuroendocrine prostate cancer. Nat. Commun..

[5] Wang Z., Yang B., Zhang M., Guo W., Wu Z., Wang Y., Jia L., Li S., Xie W., Yang D. (2018). lncRNA epigenetic landscape analysis Identifies EPIC1 as an oncogenic lncRNA that Interacts with MYC and promotes cell-cycle progression in cancer. Cancer Cell.

[6] Ghafouri-Fard S., Esmaeili M., Taheri M. (2020). H19 lncRNA: roles in tumorigenesis. Biomed. Pharmacother..

[7] Ng M., Heckl D., Klusmann J.H. (2019). The regulatory roles of long noncoding RNAs in acute myeloid leukemia. Front. Oncol..

[8] Qiu Y., Xu M., Huang S. (2021). Long noncoding RNAs: emerging regulators of normal and malignant hematopoiesis. Blood.

[9] Luo H., Zhu G., Xu J., Lai Q., Yan B., Guo Y., Fung T.K., Zeisig B.B., Cui Y., Zha J. (2019). HOTTIP lncRNA promotes hematopoietic stem cell self-renewal leading to AML-like disease in mice. Cancer Cell.

[10] Peng W.X., Koirala P., Mo Y.Y. (2017). LncRNA-mediated regulation of cell signaling in cancer. Oncogene.

[11] Statello L., Guo C.J., Chen L.L., Huarte M. (2021). Gene regulation by long non-coding RNAs and its biological functions. Nat. Rev. Mol. Cell Biol..

[12] Thierry-Mieg D., Thierry-Mieg J. (2006). AceView: a comprehensive cDNA-supported gene and transcripts annotation. Genome Biol..

[13] Graham L.D., Pedersen S.K., Brown G.S., Ho T., Kassir Z., Moynihan A.T., Vizgoft E.K., Dunne R., Pimlott L., Young G.P. (2011). Colorectal Neoplasia Differentially Expressed (CRNDE), a novel gene with elevated expression in colorectal adenomas and adenocarcinomas. Genes Cancer.

[14] Ma X., Zhang W., Zhang R., Li J., Li S., Ma Y., Jin W., Wang K. (2019). Overexpressed long noncoding RNA CRNDE with distinct alternatively spliced isoforms in multiple cancers. Front. Med..

[15] Ellis B.C., Molloy P.L., Graham L.D. (2012). CRNDE: a long non-coding RNA involved in CanceR, neurobiology, and DEvelopment. Front. Genet..

[16] Ma X., Zhang W., Zhao M., Li S., Jin W., Wang K. (2020). Oncogenic role of lncRNA CRNDE in acute promyelocytic leukemia and NPM1-mutant acute myeloid leukemia. Cell Death Discov..

[17] Ellis B.C., Kassir Z., Molloy P.L., Graham L.D. (2012). CRNDE, a novel non-coding RNA (ncRNA) gene with elevated expression in colorectal neoplasia. Hum. Genome Meet..

[18] Szafron L.M., Balcerak A., Grzybowska E.A., Pienkowska-Grela B., Felisiak-Golabek A., Podgorska A., Kulesza M., Nowak N., Pomorski P., Wysocki J. (2015). The novel gene CRNDE encodes a nuclear peptide (CRNDEP) which is overexpressed in highly proliferating tissues. PLoS One.

[19] Jiang H., Wang Y., Ai M., Wang H., Duan Z., Wang H., Zhao L., Yu J., Ding Y., Wang S. (2017). Long noncoding RNA CRNDE stabilized by hnRNPUL2 accelerates cell proliferation and migration in colorectal carcinoma via activating Ras/MAPK signaling pathways. Cell Death Dis..

[20] Han P., Li J.W., Zhang B.M., Lv J.C., Li Y.M., Gu X.Y., Yu Z.W., Jia Y.H., Bai X.F., Li L. (2017). The lncRNA CRNDE promotes colorectal cancer cell proliferation and chemoresistance via miR-181a-5p-mediated regulation of Wnt/beta-catenin signaling. Mol. Cancer.

[21] Zhang J.J., Fan L.P. (2019). Long non-coding RNA CRNDE enhances cervical cancer progression by suppressing PUMA expression. Biomed. Pharmacother..

[22] Yu B., Ye X., Du Q., Zhu B., Zhai Q., Li X.X. (2017). The long non-Coding RNA CRNDE promotes colorectal carcinoma progression by competitively binding miR-217 with TCF7L2 and enhancing the Wnt/beta-Catenin signaling pathway. Cell Physiol. Biochem..

[23] Wang H., Ke J., Guo Q., Barnabo Nampoukime K.P., Yang P., Ma K. (2018). Long non-coding RNA CRNDE promotes the proliferation, migration and invasion of hepatocellular carcinoma cells through miR-217/MAPK1 axis. J. Cell Mol Med..

[24] Wang G., Pan J., Zhang L., Wei Y., Wang C. (2017). Long non-coding RNA CRNDE sponges miR-384 to promote proliferation and metastasis of pancreatic cancer cells through upregulating IRS1. Cell Prolif..

[25] Ding J., Li J., Wang H., Tian Y., Xie M., He X., Ji H., Ma Z., Hui B., Wang K. (2017). Long noncoding RNA CRNDE promotes colorectal cancer cell proliferation via epigenetically silencing DUSP5/CDKN1A expression. Cell Death Dis..

[26] Sun J., Jia H., Bao X., Wu Y., Zhu T., Li R., Zhao H. (2021). Tumor exosome promotes Th17 cell differentiation by transmitting the lncRNA CRNDE-h in colorectal cancer. Cell Death Dis..

[27] Zhang J., Yin M., Peng G., Zhao Y. (2018). CRNDE: an important oncogenic long non-coding RNA in human cancers. Cell Prolif..

[28] Xie S.C., Zhang J.Q., Jiang X.L., Hua Y.Y., Xie S.W., Qin Y.A., Yang Y.J. (2020). LncRNA CRNDE facilitates epigenetic suppression of CELF2 and LATS2 to promote proliferation, migration and chemoresistance in hepatocellular carcinoma. Cell Death Dis..

[29] Tang Z., Li C., Kang B., Gao G., Li C., Zhang Z. (2017). GEPIA: a web server for cancer and normal gene expression profiling and interactive analyses. Nucleic Acids Res..

[30] Liu T., Zhang X., Gao S., Jing F., Yang Y., Du L., Zheng G., Li P., Li C., Wang C. (2016). Exosomal long noncoding RNA CRNDE-h as a novel serum-based biomarker for diagnosis and prognosis of colorectal cancer. Oncotarget.

[31] Liang C., Zhang B., Ge H., Xu Y., Li G., Wu J. (2018). Long non-coding RNA CRNDE as a potential prognostic biomarker in solid tumors: a meta-analysis. Clin. Chim. Acta.

[32] He T.Y., Li S.H., Huang J., Gong M., Li G. (2019). Prognostic value of long non-coding RNA CRNDE in gastrointestinal cancers: a meta-analysis. Cancer Manag. Res..

[33] Hongzhen Z., Yanyu L., Xuexiang L., Meiyu D., Xiaoli C., Yun G., Jingfan C., Shengming D. (2019). The diagnostic and prognostic significance of long non-coding RNA CRNDE in pan-cancer based on TCGA, GEO and comprehensive meta-analysis. Pathol. Res. Pract..

[34] Zhang M., Gao C., Yang Y., Li G., Dong J., Ai Y., Chen N., Li W. (2018). Long noncoding RNA CRNDE/PRC2 participated in the radiotherapy resistance of human lung adenocarcinoma through targeting p21 expression. Oncol. Res..

[35] Lu Y., Sha H., Sun X., Zhang Y., Wu Y., Zhang J., Zhang H., Wu J., Feng J. (2020). CRNDE: an oncogenic long non-coding RNA in cancers. Cancer Cell Int..

[36] Ding Q., Mo F., Cai X., Zhang W., Wang J., Yang S., Liu X. (2020). LncRNA CRNDE is activated by SP1 and promotes osteosarcoma proliferation, invasion, and epithelial-mesenchymal transition via Wnt/beta-catenin signaling pathway. J. Cell Biochem..

[37] Liu X.X., Xiong H.P., Huang J.S., Qi K., Xu J.J. (2017). Highly expressed long non-coding RNA CRNDE promotes cell proliferation through PI3K/AKT signalling in non-small cell lung carcinoma. Clin. Exp. Pharmacol. Physiol..

[38] Gao H., Song X., Kang T., Yan B., Feng L., Gao L., Ai L., Liu X., Yu J., Li H. (2017). Long noncoding RNA CRNDE functions as a competing endogenous RNA to promote metastasis and oxaliplatin resistance by sponging miR-136 in colorectal cancer. Onco Targets Ther..

[39] Ellis B.C., Graham L.D., Molloy P.L. (2014). CRNDE, a long non-coding RNA responsive to insulin/IGF signaling, regulates genes involved in central metabolism. Biochim. Biophys. Acta.

[40] Li H., Li Q., Guo T., He W., Dong C., Wang Y. (2017). LncRNA CRNDE triggers inflammation through the TLR3-NF-kappaB-Cytokine signaling pathway. Tumour Biol..

[41] Kiang K.M., Zhang X.Q., Zhang G.P., Li N., Cheng S.Y., Poon M.W., Pu J.K., Lui W.M., Leung G.K. (2017). CRNDE expression positively correlates with EGFR activation and modulates glioma cell growth. Target Oncol..

[42] Wang Y., Wang Y., Li J., Zhang Y., Yin H., Han B. (2015). CRNDE, a long-noncoding RNA, promotes glioma cell growth and invasion through mTOR signaling. Cancer Lett..

[43] Zheng J., Li X.D., Wang P., Liu X.B., Xue Y.X., Hu Y., Li Z., Li Z.Q., Wang Z.H., Liu Y.H. (2015). CRNDE affects the malignant biological characteristics of human glioma stem cells by negatively regulating miR-186. Oncotarget.

[44] Zheng J., Liu X., Wang P., Xue Y., Ma J., Qu C., Liu Y. (2016). CRNDE promotes malignant progression of glioma by attenuating miR-384/PIWIL4/STAT3 axis. Mol. Ther..

[45] Li D.X., Fei X.R., Dong Y.F., Cheng C.D., Yang Y., Deng X.F., Huang H.L., Niu W.X., Zhou C.X., Xia C.Y. (2017). The long non-coding RNA CRNDE acts as a ceRNA and promotes glioma malignancy by preventing miR-136-5p-mediated downregulation of Bcl-2 and Wnt2. Oncotarget.

[46] Tang Q., Zheng X., Zhang J. (2018). Long non-coding RNA CRNDE promotes heptaocellular carcinoma cell proliferation by regulating PI3K/Akt/beta-catenin signaling. Biomed. Pharmacother..

[47] Zhu L., Yang N., Du G., Li C., Liu G., Liu S., Xu Y., Di Y., Pan W., Li X. (2018). LncRNA CRNDE promotes the epithelial-mesenchymal transition of hepatocellular carcinoma cells via enhancing the Wnt/beta-catenin signaling pathway. J. Cell Biochem..

[48] Chen Z., Yu C., Zhan L., Pan Y., Chen L., Sun C. (2016). LncRNA CRNDE promotes hepatic carcinoma cell proliferation, migration and invasion by suppressing miR-384. Am. J. Cancer Res..

[49] Ji D., Jiang C., Zhang L., Liang N., Jiang T., Yang B., Liang H. (2019). LncRNA CRNDE promotes hepatocellular carcinoma cell proliferation, invasion, and migration through regulating miR-203/BCAT1 axis. J. Cell Physiol..

[50] Zhu L., Liu Y., Chen Q., Yu G., Chen J., Chen K., Yang N., Zeng T., Yan S., Huang A. (2018). Long-noncoding RNA colorectal neoplasia differentially expressed gene as a potential target to upregulate the expression of IRX5 by miR-136-5P to promote oncogenic properties in hepatocellular carcinoma. Cell Physiol Biochem..

[51] Tang D., Zhao L., Peng C., Ran K., Mu R., Ao Y. (2019). LncRNA CRNDE promotes hepatocellular carcinoma progression by upregulating SIX1 through modulating miR-337-3p. J. Cell Biochem..

[52] Lin C., Xiang Y., Sheng J., Liu S., Cui M., Zhang X. (2020). Long non-coding RNA CRNDE promotes malignant progression of hepatocellular carcinoma through the miR-33a-5p/CDK6 axis. J. Physiol. Biochem..

[53] Chen L.J., Yuan M.X., Ji C.Y., Zhang Y.B., Peng Y.M., Zhang T., Gao H.Q., Sheng X.Y., Liu Z.Y., Xie W.X. (2020). Long non-coding RNA CRNDE regulates angiogenesis in hepatoblastoma by targeting the MiR-203/VEGFA Axis. Pathobiology.

[54] Li Z., Wu G., Li J., Wang Y., Ju X., Jiang W. (2020). lncRNA CRNDE promotes the proliferation and metastasis by acting as sponge miR-539-5p to regulate POU2F1 expression in HCC. BMC Cancer.

[55] Fan Y.F., Yu Z.P., Cui X.Y. (2019). lncRNA colorectal neoplasia differentially expressed (CRNDE) promotes proliferation and inhibits apoptosis in non-small cell lung cancer cells by regulating the miR-641/CDK6 Axis. Med. Sci. Monit..

[56] Jing H., Xia H., Qian M., Lv X. (2019). Long noncoding RNA CRNDE promotes non-small cell lung cancer progression via sponging microRNA-338-3p. Biomed. Pharmacother..

[57] Bai X., Wang W., Zhao P., Wen J., Guo X., Shen T., Shen J., Yang X. (2020). LncRNA CRNDE acts as an oncogene in cervical cancer through sponging miR-183 to regulate CCNB1 expression. Carcinogenesis.

[58] Yang H.Y., Huang C.P., Cao M.M., Wang Y.F., Liu Y. (2018). Long non-coding RNA CRNDE may be associated with poor prognosis by promoting proliferation and inhibiting apoptosis of cervical cancer cells through targeting PI3K/AKT. Neoplasma.

[59] Ren L., Yang S., Cao Q., Tian J. (2021). CRNDE contributes cervical cancer progression by regulating miR-4262/ZEB1 Axis. Onco Targets Ther..

[60] Huan J., Xing L., Lin Q., Xui H., Qin X. (2017). Long noncoding RNA CRNDE activates Wnt/beta-catenin signaling pathway through acting as a molecular sponge of microRNA-136 in human breast cancer. Am. J. Transl Res..

[61] Ren Y., He W., Chen W., Ma C., Li Y., Zhao Z., Gao T., Ni Q., Chai J., Sun M. (2019). CRNDE promotes cell tongue squamous cell carcinoma cell growth and invasion through suppressing miR-384. J. Cell Biochem..

[62] Sun H., He L., Ma L., Lu T., Wei J., Xie K., Wang X. (2017). LncRNA CRNDE promotes cell proliferation, invasion and migration by competitively binding miR-384 in papillary thyroid cancer. Oncotarget.

[63] Sun X.H., Fan W.J., An Z.J., Sun Y. (2020). Inhibition of long noncoding RNA CRNDE increases chemosensitivity of medulloblastoma cells by targeting miR-29c-3p. Oncol. Res..

[64] Zhang Y., Lan X., Wang Y., Liu C., Cui T. (2020). CRNDE mediates the viability and epithelial-mesenchymal transition of renal cell carcinoma via miR-136-5p. J. Recept Signal. Transduct. Res..

[65] Chen J.H., Tong W., Pu X.F., Wang J.Z. (2020). Long noncoding RNA CRNDE promotes proliferation, migration and invasion in prostate cancer through miR-101/Rap1A. Neoplasma.

[66] Li Z., Tang Y., Xing W., Dong W., Wang Z. (2018). LncRNA, CRNDE promotes osteosarcoma cell proliferation, invasion and migration by regulating Notch1 signaling and epithelial-mesenchymal transition. Exp. Mol. Pathol..

[67] Mulati M., Kobayashi Y., Takahashi A., Numata H., Saito M., Hiraoka Y., Ochi H., Sato S., Ezura Y., Yuasa M. (2020). The long noncoding RNA Crnde regulates osteoblast proliferation through the Wnt/beta-catenin signaling pathway in mice. Bone.

[68] Xu L., Zhang Y., Zhao Z., Chen Z., Wang Z., Xu S., Zhang X., Liu T., Yu S. (2018). The long non-coding RNA CRNDE competed endogenously with miR-205 to promote proliferation and metastasis of melanoma cells by targeting CCL18. Cell Cycle.

[69] Meng Y.B., He X., Huang Y.F., Wu Q.N., Zhou Y.C., Hao D.J. (2017). Long noncoding RNA CRNDE promotes multiple myeloma cell growth by suppressing miR-451. Oncol. Res..

[70] David A., Zocchi S., Talbot A., Choisy C., Ohnona A., Lion J., Cuccuini W., Soulier J., Arnulf B., Bories J.C. (2020). The long non-coding RNA CRNDE regulates growth of multiple myeloma cells via an effect on IL6 signalling. Leukemia.

[71] Wang W., Wu F., Ma P., Gan S., Li X., Chen L., Sun L., Sun H., Jiang Z., Guo F. (2020). LncRNA CRNDE promotes the progression of B-cell precursor acute lymphoblastic leukemia by targeting the miR-345-5p/CREB Axis. Mol. Cells.

[72] Ni J., Hong J., Li Q., Zeng Q., Xia R. (2021). Long non-coding RNA CRNDE suppressing cell proliferation is regulated by DNA methylation in chronic lymphocytic leukemia. Leuk. Res..

[73] Arnold M., Sierra M.S., Laversanne M., Soerjomataram I., Jemal A., Bray F. (2017). Global patterns and trends in colorectal cancer incidence and mortality. Gut.

[74] Niu L., Yang W., Duan L., Wang X., Li Y., Xu C., Liu C., Zhang Y., Zhou W., Liu J. (2021). Biological implications and clinical potential of metastasis-related miRNA in colorectal cancer. Mol. Ther. Nucleic Acids.

[75] Hanif F., Muzaffar K., Perveen K., Malhi S.M., Simjee Sh U. (2017). Glioblastoma multiforme: a review of its epidemiology and pathogenesis through clinical presentation and treatment. Asian Pac. J. Cancer Prev..

[76] Jing S.Y., Lu Y.Y., Yang J.K., Deng W.Y., Zhou Q., Jiao B.H. (2016). Expression of long non-coding RNA CRNDE in glioma and its correlation with tumor progression and patient survival. Eur. Rev. Med. Pharmacol. Sci..

[77] Chen Y., Wu J.J., Lin X.B., Bao Y., Chen Z.H., Zhang C.R., Cai Z., Zhou J.Y., Ding M.H., Wu X.J. (2015). Differential lncRNA expression profiles in recurrent gliomas compared with primary gliomas identified by microarray analysis. Int. J. Clin. Exp. Med..

[78] Yang J.D., Hainaut P., Gores G.J., Amadou A., Plymoth A., Roberts L.R. (2019). A global view of hepatocellular carcinoma: trends, risk, prevention and management. Nat. Rev. Gastroenterol. Hepatol..

[79] Singal A.G., Lampertico P., Nahon P. (2020). Epidemiology and surveillance for hepatocellular carcinoma: new trends. J. Hepatol..

[80] Zhao Y., Li Y., Liu W., Xing S., Wang D., Chen J., Sun L., Mu J., Liu W., Xing B. (2020). Identification of noninvasive diagnostic biomarkers for hepatocellular carcinoma by urinary proteomics. J. Proteomics.

[81] Deng L.X., Mehta N. (2020). Does hepatocellular carcinoma surveillance increase survival in at-risk populations? Patient selection, biomarkers, and barriers. Dig. Dis. Sci..

[82] Esposti D.D., Hernandez-Vargas H., Voegele C., Fernandez-Jimenez N., Forey N., Bancel B., Le Calvez-Kelm F., McKay J., Merle P., Herceg Z. (2016). Identification of novel long non-coding RNAs deregulated in hepatocellular carcinoma using RNA-sequencing. Oncotarget.

[83] Dai M., Chen S., Wei X., Zhu X., Lan F., Dai S., Qin X. (2017). Diagnosis, prognosis and bioinformatics analysis of lncRNAs in hepatocellular carcinoma. Oncotarget.

[84] Subhash S., Andersson P.O., Kosalai S.T., Kanduri C., Kanduri M. (2016). Global DNA methylation profiling reveals new insights into epigenetically deregulated protein coding and long noncoding RNAs in CLL. Clin. Epigenet,.

[85] Jones L., Wei G., Sevcikova S., Phan V., Jain S., Shieh A., Wong J.C., Li M., Dubansky J., Maunakea M.L. (2010). Gain of MYC underlies recurrent trisomy of the MYC chromosome in acute promyelocytic leukemia. J. Exp. Med..

[86] Sohal J., Phan V.T., Chan P.V., Davis E.M., Patel B., Kelly L.M., Abrams T.J., O'Farrell A.M., Gilliland D.G., Le Beau M.M. (2003). A model of APL with FLT3 mutation is responsive to retinoic acid and a receptor tyrosine kinase inhibitor, SU11657. Blood.

[87] Schessl C., Rawat V.P., Cusan M., Deshpande A., Kohl T.M., Rosten P.M., Spiekermann K., Humphries R.K., Schnittger S., Kern W. (2005). The AML1-ETO fusion gene and the FLT3 length mutation collaborate in inducing acute leukemia in mice. J. Clin. Invest..

[88] Wang Y., Zhou Q., Ma J.J. (2018). High expression of lnc-CRNDE presents as a biomarker for acute myeloid leukemia and promotes the malignant progression in acute myeloid leukemia cell line U937. Eur. Rev. Med. Pharmacol. Sci..

[89] Offidani M., Petrucci M.T. (2020). Introduction to "Immunotherapies for multiple myeloma". Pharmaceuticals.

[90] Terpos E., Ntanasis-Stathopoulos I., Gavriatopoulou M., Dimopoulos M.A. (2018). Pathogenesis of bone disease in multiple myeloma: from bench to bedside. Blood Cancer J..

[91] Matthes T., Manfroi B., Huard B. (2016). Revisiting IL-6 antagonism in multiple myeloma. Crit. Rev. Oncol. Hematol..

